# Cardiomyopathie hypertrophique néonatale de diagnostic étiologique difficile

**Published:** 2011-12-19

**Authors:** Rania Hammami, Sana Ouali, Ilyes Naffeti, Sami Hammas, Slim Kacem, Rim Gribaa, Fahmi Remedi, Essia Boughzela

**Affiliations:** 1 Service de cardiologie de Sahloul, Sousse, Tunisie

**Keywords:** Cardiomyopathie hypertrophique, étiologie, néonatal, Tunisie

## Abstract

La cardiomyopathie hypertrophique néonatale est une entité rare, hétérogène regroupant plusieurs formes cliniques et donc de diagnostic étiologique difficile. Nous rapportons l'observation d'un nouveau né issu d'une grossesse gémellaire, ayant présenté à la naissance un tableau d'insuffisance cardiaque, l’échocardiographie avait conclut à une cardiomyopathie hypertrophique obstructive. Le bilan étiologique était négatif notamment une mère non diabétique. L’évolution était favorable avec régression de l'hypertrophie 2 semaines après la naissance. L’étiologie finalement suggérée était une cardiomyopathie secondaire à l'injection anténatale de corticoïdes dans le but d'accélérer la maturation pulmonaire. L’établissement par les sociétés savantes d'un consensus de bilan étiologique minimal standard selon une chronologie bien déterminée serait d'un grand apport dans la prise en charge de cette anomalie.

## Introduction

La cardiomyopathie hypertrophique (CMH) néonatale dans sa forme régressive est une entité exceptionnelle, mal élucidée [1] rapportée dans la plupart des publications dans le cadre de diabète maternel. Le diagnostic positif est simple, basé sur l’échocardiographie mais le diagnostic étiologique est souvent difficile nécessitant le recours à plusieurs investigations biologiques et radiologiques et parfois posé sur des éléments de présomptions. L'objectif de ce travail est de rapporter un cas de cardiomyopathie hypertrophique néonatale rapidement régressive et d’étayer le bilan étiologique avec une revue de la littérature de cette entité rare.

## Cas Clinique

**Figure 1:**
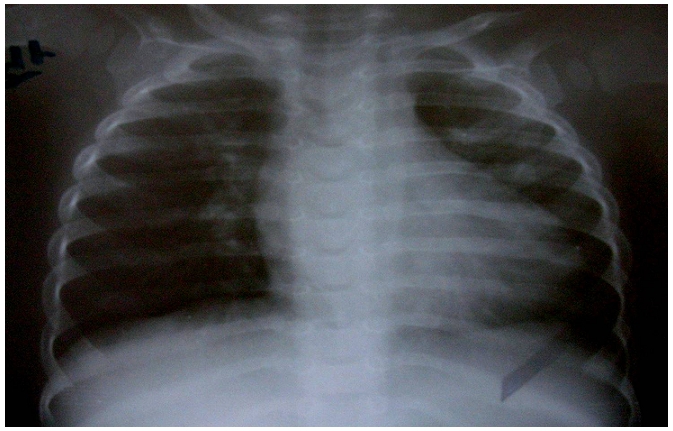
Radiographie thoracique de face; Aspect de cardiomégalie; arc inférieur gauche allongé

**Figure 2:**
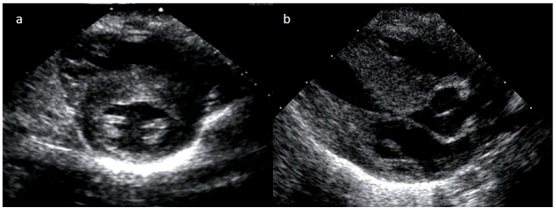
Hypertrophie ventriculaire gauche à prédominance antéro-septale en coupe para-sternale petit axe (a) avec aspect d'un mouvement systolique de la valve mitrale en coupe para-sternale grand axe (b)

**Figure 3:**
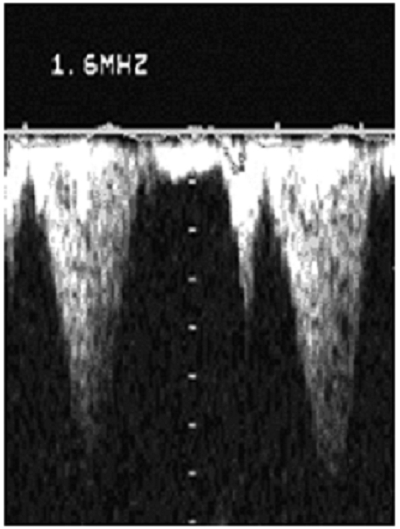
Cardiomyopathie hypertrophique obstructive; Gradient intra-ventriculaire en forme de lame de sabre en doppler continu

Le nouveau-né Z.A de sexe féminin, est issu d'une première grossesse gémellaire non suivie, menée au terme de 35 semaines d'aménorrhée, accouché par césarienne devant une toxémie gravidique. Vu que la mère avait présenté une menace d'accouchement à la 32^ème^ semaine d'aménorrhée, une maturation pulmonaire a été réalisée. La mère avait reçu deux injections de Bethamétasone par voie intramusculaire à la dose de 12 mg / injection et à 24 heures d'intervalle. A la naissance, le bébé pesait 2,5 Kg. Il était apyrétique, tachycarde à 145 battements/min et polypnéique à 65 cycles/min avec une saturation en oxygène (SaO2) à l'air ambiant de 95%. La pression artérielle était correcte (78/ 40 mmHg). L'auscultation cardiaque avait révélé un rythme régulier avec un souffle systolique méso-cardiaque irradiant aux 4 foyers. L'auscultation pulmonaire était par contre sans anomalies. Il n'y avait pas d'anomalies phénotypiques apparentes. L'examen du jumeau de sexe masculin était parfaitement normal avec un poids de naissance de 3 Kg. L’électrocardiogramme était sans anomalies évidentes à part un rythme cardiaque rapide. La radiographie thoracique avait montré une cardiomégalie avec un arc inférieur gauche allongé (Figure 1). L’échocardiographie avait révélé un ventricule gauche hypertrophié de façon excentrée, avec prédominance de celle-ci sur la paroi antéro-septale (épaisseur septale de 13 mm et épaisseur de la paroi postérieure de 9 mm) et une fraction de raccourcissement de 50% (Figure 2). Il y avait une accélération du flux au niveau de la chambre de chasse avec un gradient maximum intra VG élevé à 46 mmHg (Figure 3). La grande valve mitrale présentait un mouvement antérieur systolique mais sans fuite mitrale. Aucune autre cardiopathie congénitale n’était identifiée à l’échocardiographie, notamment pas de coarctation de l'aorte ni de sténose aortique congénitale. Le diagnostic de cardiomyopathie hypertrophique obstructive a été retenu et le bébé était mis sous Lasilix par voie orale à la dose de 1,5 mg/Kg/jour. Une enquête étiologique démarrée n'avait pas montré d'antécédents cardiaques dans la famille ni de cas similaires. L’échocardiographie était normale chez les parents ainsi que le jumeau. Il n'y avait pas d'antécédents de diabète chez la mère et le chiffre d'Hb glyquée était de 6,5%. Le caryotype du bébé était normal. Une série de dosages biologiques a été réalisée et revenue normale: hémoglobine sanguine, dosage de la carnitine totale et de la carnitine libre dans le sang, dosage des catécholamines urinaires, dosage de l'activité a glucosidase et dosage de la créatine kinase. L'examen anatomopathologique d'une biopsie musculaire s'est également révélé normal.

Au cours de l’évolution, une amélioration clinique nette a été observée avec régression du souffle cardiaque et une prise de poids convenable. Une échocardiographie de contrôle réalisée après deux semaines avait montré une régression nette de l'hypertrophie avec une épaisseur respective des parois septale et postérieure de 8mm et 5 mm et un gradient maximal intra-VG à 20 mmHg. La décision alors était d'arrêter les explorations et de surveiller le bébé, un contrôle échocardiographique à 1, 3 et 6 mois était sans anomalies avec une croissance strictement normale.

Après revue de la littérature, l’étiologie retenue pour cette hypertrophie cardiaque néonatale transitoire était une réaction secondaire aux corticoïdes injectés dans le but d'accélérer la maturation pulmonaire. Un éventuel effet potentiel de la décharge catécholérgique maternelle au cours de la toxémie gravidique ne serait pas exclu.

## Discussion

La cardiomyopathie hypertrophique néonatale est une entité rare, rapportée dans la littérature sous forme de cas cliniques isolés, elle est définie par une hypertrophie ventriculaire gauche asymétrique à prédominance septale avec une fonction systolique normale [2]. La diversité étiologique de la CMH rend compte de la difficulté de sa prise en charge (Tableau 1). Dans le registre épidémiologique de CMH infantile [1], 69,2% des CMH diagnostiquées avant l’âge d'une année sont considérées idiopathiques.

**Tableau 1:** Classification de la cardiomyopathie hypertrophique

CMH obstructive/ non obstructive
CMH primaire/secondaire
CMH d'origine métabolique: déficit en carnitine, déficit enzymatique (GSDIII), maladie de Fabry, Fucosidose type I, syndrome de Hynter, manosidose, syndrome de Huler, déficit en sélénium, enfant de mère diabétique, excès de corticoïdes ou catécholamines…
CMH syndromique: syndrome de Bechwith-Wiedman, syndrome cardio-facial-cutané, ataxie de Freiderich, syndrome de Noonan
CMH associée à des myopathies: myopathie mitochondriale, déficit en cytochrome c réductase, myopathie histiocytoide
CMH idiopathique

La forme de CMH chez les prématurés, secondaire à un traitement par des corticoïdes dans le cadre de prévention de la dysplasie broncho-alvéolaire est bien décrite dans la littérature [4,5] mais les formes secondaires à une injection anténatale de corticoïdes sont très exceptionnelles et limitées à quelques observations [6]. Cette entité se présente souvent sous une forme clinique bien tolérée, de régression rapide (au bout de quelques semaines) à la différence des formes associées à un diabète maternel où la restitution *ad integrum* se fait parfois au bout d'une année. Kalid et al [6] ont rapporté 3 cas de cardiomyopathie hypertrophique transitoire chez des nouveaux nés dont les mères avaient reçu des doses répétitives de corticoïdes anténatales (5 à 16 cures), les auteurs ont considéré que l'apparition d'hypertrophie ventriculaire dépend en fait de la dose et de la durée d'injection de corticoïdes chez la mère avant l'accouchement. Cependant, et au vue de notre observation on pense que même des doses faibles pourraient causer cette pathologie puisque la mère n'a reçu qu'une seule cure de Béthamétasone. L'association à des conditions de stress (toxémie gravidique) aves décharges de catécholamines pourrait potentialiser l'effet des corticoïdes sur le muscle cardiaque et favoriser l'hypertrophie des cellules musculaires cardiaques.

Dans notre observation, la survenue chez un seul bébé des jumeaux soumis aux mêmes conditions suggèrent de plus un facteur génétique d'autant plus qu'il s'agit d'une grossesse bi-choriale bi-amniotique. Le faible poids du bébé malade est probablement un autre facteur favorisant. Tous ces indices pourraient suggérer qu'il s'agit d'une entité multifactorielle.

En effet, jusqu′à nos jours, l’étiopathogènie de la CMH secondaire aux corticoïdes reste peu identifiée. Certains travaux expérimentaux élaborés sur des cœurs de rats suggèrent un rôle important d'une transcription du gène SGK1 ce qui génère une croissance excessive de la taille des cellules, de la synthèse protéique et de l'organisation des sarcomères [7]. D'autres considèrent que cette transcription favorise plutôt l'expression de la chaine lourde de l'a myosine [8]. La récurrence de cette entité chez des bébés souvent prématurés de faible poids pourrait offrir un autre indice des mécanismes moléculaires impliqués.

## Conclusion

La CMH néonatale est une pathologie rare, hétérogène, de diagnostic étiologique difficile. Sa régression rapide doit faire penser à une cause métabolique en particulier le diabète maternel, ou une cause iatrogène médicamenteuse notamment les corticoïdes. Un bilan étiologique exhaustif s'impose toujours mais c'est un bilan couteux et lent, un consensus d'un bilan minimal établi par les sociétés savantes pourrait améliorer et faciliter la prise en charge.
